# Ventilation–perfusion heterogeneity measured by the multiple inert gas elimination technique is minimally affected by intermittent breathing of 100% O_2_

**DOI:** 10.14814/phy2.14488

**Published:** 2020-07-07

**Authors:** Ann R. Elliott, Abhilash S. Kizhakke Puliyakote, Vincent Tedjasaputra, Beni Pazár, Harrieth Wagner, Rui C. Sá, Jeremy E. Orr, G. Kim Prisk, Peter D. Wagner, Susan R. Hopkins

**Affiliations:** ^1^ Department of Medicine University of California San Diego La Jolla CA USA; ^2^ The Pulmonary Imaging Laboratory University of California San Diego La Jolla CA USA; ^3^ Department of Radiology University of California San Diego La Jolla CA USA

**Keywords:** hyperoxia, magnetic resonance imaging, pulmonary perfusion distribution, pulmonary ventilation distribution, specific ventilation imaging, ventilation‐perfusion ratio

## Abstract

Proton magnetic resonance (MR) imaging to quantify regional ventilation–perfusion ( ${\dot{V}}_{A}/\dot{Q}$) ratios combines specific ventilation imaging (SVI) and separate proton density and perfusion measures into a composite map. Specific ventilation imaging exploits the paramagnetic properties of O_2_, which alters the local MR signal intensity, in an F_I_O_2_‐dependent manner. Specific ventilation imaging data are acquired during five wash‐in/wash‐out cycles of breathing 21% O_2_ alternating with 100% O_2_ over ~20 min. This technique assumes that alternating F_I_O_2_ does not affect ${\dot{V}}_{A}/\dot{Q}$ heterogeneity, but this is unproven. We tested the hypothesis that alternating F_I_O_2_ exposure increases ${\dot{V}}_{A}/\dot{Q}$ mismatch in nine patients with abnormal pulmonary gas exchange and increased ${\dot{V}}_{A}/\dot{Q}$ mismatch using the multiple inert gas elimination technique (MIGET).The following data were acquired (a) breathing air (baseline), (b) breathing alternating air/100% O_2_ during an emulated‐SVI protocol (eSVI), and (c) 20 min after ambient air breathing (recovery). MIGET heterogeneity indices of shunt, deadspace, ventilation versus ${\dot{V}}_{A}/\dot{Q}$ ratio, LogSD $\dot{V}$, and perfusion versus ${\dot{V}}_{A}/\dot{Q}$ ratio, LogSD $\dot{Q}$ were calculated. LogSD $\dot{V}$ was not different between eSVI and baseline (1.04 ± 0.39 baseline, 1.05 ± 0.38 eSVI, *p* = .84); but was reduced compared to baseline during recovery (0.97 ± 0.39, *p* = .04). There was no significant difference in LogSD $\dot{Q}$ across conditions (0.81 ± 0.30 baseline, 0.79 ± 0.15 eSVI, 0.79 ± 0.20 recovery; *p* = .54); Deadspace was not significantly different (*p* = .54) but shunt showed a borderline increase during eSVI (1.0% ± 1.0 baseline, 2.6% ± 2.9 eSVI; *p* = .052) likely from altered hypoxic pulmonary vasoconstriction and/or absorption atelectasis. Intermittent breathing of 100% O_2_ does not substantially alter ${\dot{V}}_{A}/\dot{Q}$ matching and if SVI measurements are made after perfusion measurements, any potential effects will be minimized.

## Graphical abstract

Intermittent hyperoxia is used in proton magnetic resonance imaging to evaluate ventilation as part of measuring ventilation‐perfusion mismatch, but it may alter the underlying ventilation–perfusion distribution, especially when ventilation‐perfusion mismatch is increased. We used the multiple inert gas elimination technique to evaluate the effect of breathing alternating 2‐min‐100% oxygen with 2‐min‐21% oxygen on ventilation‐perfusion distributions in subjects with increased ventilation‐perfusion mismatch. There was a borderline increase in shunt, but no other significant effect on ventilation‐perfusion heterogeneity, suggesting that oxygen can be used to evaluate the ventilation‐perfusion distribution especially if perfusion images are acquired before oxygen is used in measuring ventilation.

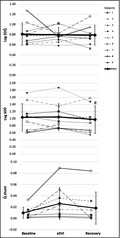

## 1 INTRODUCTION

Many lung diseases are characterized by an increase in ventilation–perfusion ( ${\dot{V}}_{A}/\dot{Q}$) mismatch, which impairs pulmonary gas exchange. Our laboratory has developed a proton magnetic resonance imaging (MRI) technique, to measure regional ventilation‐perfusion mismatch, which combines specific ventilation imaging (SVI) with proton density (Holverda et al., 2011; Theilmann et al., 2009) and perfusion (Bolar et al., 2006) images to give regional ${\dot{V}}_{A}/\dot{Q}$ ratios (Henderson et al., 2013; Sá et al., 2017).

Specific ventilation imaging noninvasively measures regional specific ventilation (Sá et al., 2010, 2014). During SVI, the subject breathes 20 breaths of 100% O_2_ (~2 min, washin) followed by 20 breaths of air (~2 min, washout) and repeats this alternating block five times. Oxygen is paramagnetic; by breathing 100% O_2_ and using an appropriate magnetic resonance sequence, inhaled O_2_ is used as an MRI contrast agent (Sá et al., 2010, 2014). By analyzing the time course of the change in MR signal intensity on a voxel by voxel basis, a specific ventilation map is constructed (Sá et al., 2010, 2014), and then combined with density and perfusion measures.

Although it is a common tool in pulmonary function testing, (Comroe & Fowler, 1951; Fowler, 1949; Lewis, Evans, & Jalowayski, 1978; Martin, Tsunoda, & Young, 1974; Robinson et al., 2013; Verbanck et al., 1997, 1998), breathing hyperoxic gas could affect the underlying pulmonary physiology and ${\dot{V}}_{A}/\dot{Q}$ matching. For example, O_2_ breathing can alter regional blood flow and ventilation predominately by either the release of hypoxic pulmonary vasoconstriction and/or absorption atelectasis (Dawson, 1969, 1984; Lee & Read, 1967; Morrell, Nijran, Biggs, & Seed, 1994), but other mechanisms can also modulate these processes. Hyperoxia can either directly or indirectly alter bronchomotor tone (Astin & Penman, 1967; Libby, Briscoe, & King, 1981), which in turn potentially increases ${\dot{V}}_{A}/\dot{Q}$ heterogeneity (Robinson, Freiberg, Regnis, & Young, 2000; Sassoon, Hassell, & Mahutte, 1987). Additionally, hyperoxia, due to its effects on blood flow and/or ventilatory drive (Aubier et al., 1980; Robinson et al., 2000; Sassoon et al., 1987) may alter local alveolar CO_2_ which may in turn affect local lung mechanics and ventilation (Emery, Eveland, Kim, Hildebrandt, & Swenson, 2007; Emery, Eveland, Min, Hildebrandt, & Swenson, 2013; Traystman, Batra, & Menkes, 1976), and thus the underlying ${\dot{V}}_{A}/\dot{Q}$ distribution.

The effect of intermittent hyperoxia, as used in proton MRI evaluation of ${\dot{V}}_{A}/\dot{Q}$ relationships, on ${\dot{V}}_{A}/\dot{Q}$ mismatch is unknown. Previously we showed that in healthy subjects, exposure to either hyperoxia or hypoxia did not affect ventilation heterogeneity (Hopkins, Elliott, Prisk, & Darquenne, 2017), but in healthy subjects any underlying ${\dot{V}}_{A}/\dot{Q}$ mismatch is expected to be minimal and thus potential changes may be minimized. In the present study, we evaluated the effect of an emulated SVI (eSVI) O_2_ breathing protocol on ${\dot{V}}_{A}/\dot{Q}$ heterogeneity in nine subjects with a clinical diagnosis of COPD. This subject population was chosen because they could reasonably be expected to show ${\dot{V}}_{A}/\dot{Q}$ mismatch at baseline. We hypothesized that an eSVI protocol would increase ${\dot{V}}_{A}/\dot{Q}$ heterogeneity consistent with underlying effects of O_2_ either on hypoxic pulmonary vasoconstriction or by altering the ventilation distribution or a combination of both. To test this, we measured ${\dot{V}}_{A}/\dot{Q}$ mismatch with the multiple inert gas elimination technique (MIGET) (Hlastala, 1984; Hlastala & Robertson, 1978; Roca & Wagner, 1994; Wagner, Naumann, & Laravuso, 1974), a well‐established method for quantitatively assessing ${\dot{V}}_{A}/\dot{Q}$ matching.

## 2 METHODS

### 2.1 Subjects

This study was approved by the University of California, San Diego's Human Subjects’ Research Protection Program. Subjects were recruited via advertisements, pulmonary clinics, and existing research databases. Recruited subjects all had a diagnosis of COPD made by their personal physician, and all had a significant history of cigarette smoking (20 ± 12 pack‐years). All subjects gave written informed consent prior to participating. Recruited subjects were screened with a medical questionnaire for assessment of exclusion criteria that included any history of significant illness aside from a diagnosis of COPD or a current history of a recent exacerbation. Nine subjects, seven males and two females, between 49 and 70 years old participated. Subjects remained on their daily medication such as long‐acting bronchodilators and inhaled steroids for the study. Eight subjects completed the entire protocol; and one individual, subject #7, declined to complete the recovery portion of the study because of time constraints.

### 2.2 Overall study design

**FIGURE 1:**
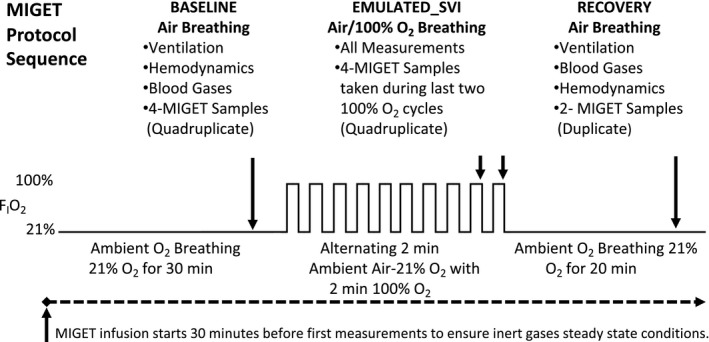
MIGET protocol sequence for data collection and sampling

On the day of the study, all subjects underwent spirometry using an EasyOne spirometer (ndd Medical Technologies, Zurich, Switzerland). To assess the influences of alternating F_I_O_2_ concentrations on ${\dot{V}}_{A}/\dot{Q}$ heterogeneity, subjects were studied using MIGET during three conditions: (a) steady‐state quiet breathing of ambient air, baseline; (b) at the end of eSVI which consisted of alternating breathing air for 2 min followed by 2 min of 100% O_2_ for a total of 20 min; and (c) after a 20‐min period of air breathing, recovery (Figure 1). During the eSVI section of the protocol, to maximize the chance of seeing potential effects of F_I_O_2_ on the ${\dot{V}}_{A}/\dot{Q}$ distribution, data and samples were acquired during the last two blocks (14–16 and 18–20 min) of 100% O_2_ breathing.

### 2.3 MIGET protocol

#### 2.3.1 Experimental setup

The subjects were supine for the study duration duplicating the necessary posture of subjects during SVI MRI data acquisition. The nondominant arm radial artery was catheterized using a 20 gauge, 1.25” catheter, for the collection of arterial blood samples, and for direct arterial pressure monitoring. A peripheral vein from the contralateral arm was catheterized with an 18 gauge, 2” catheter for the infusion of 5% dextrose solution containing the inert gas mixture. Body temperature was measured using an oral thermometer. ECG and cardiac output were monitored with a tetrapolar impedance cardiograph (model BioZ ICG, CardioDynamics), which was previously validated against direct Fick measures of cardiac output (Yung, Fedullo, Kinninger, Johnson, & Channick, 2004).

#### 2.3.2 Multiple inert gas elimination technique

The multiple inert gas elimination technique has been previously described in detail (Wagner, Laravuso, Uhl, & West, 1974; Wagner, Saltzman, & West, 1974), therefore is only briefly described here. Maintaining sterility, the six usual MIGET inert gases, (sulfur hexafluoride, ethane, cyclopropane, enflurane, ether, and acetone) were dissolved in 5% dextrose. This solution was continuously infused intravenously for the entire study duration at a rate of 2–5 ml/min. The rate was selected based on the subject's minute ventilation, at approximately 1 ml/min per 2 L/min ventilation, and has been shown to provide adequate signal to noise in the measurement of all inert gas levels. The infusion was started 30 min prior to baseline measurements to ensure steady‐state conditions for inert gases. From the last 10 min onward, the subject breathed on a mouthpiece into a heated nonrebreathing valve (model 2700, Hans Rudolph Inc) that was connected to a heated (~45°C) low resistance expired gas mixing box. Steady‐state conditions were confirmed by ensuring stable inert gas infusion, ventilation, and cardiac output prior to and during measurements.

#### 2.3.3 Sampling protocol

For each condition (baseline, eSVI, recovery), mixed expired O_2_ and CO_2_ concentrations were continuously sampled and measured from the mixing box using a mass spectrometer (Perkin Elmer, MGA 1100) and recorded using data acquisition software (LabView, National Instruments), while simultaneously measuring minute ventilation ( ${\dot{V}}_{E}$) using a calibrated gas rotameter (100 Liter, BOC), and mixing box temperature. For each condition, the data were averaged to calculate O_2_ consumption, carbon dioxide production, and the respiratory exchange ratio ( $\dot{V}$O_2_, $\dot{V}$CO_2_, RER, respectively). Four arterial blood samples (5–7 ml each), and four time‐aligned mixed expired gas samples (20 ml each) were collected for quadruplicate measurement of inert gas levels, while simultaneously measuring cardiac output. Immediately after each inert gas sample, 2 ml of arterial blood gas sample was drawn into a preheparinized 3‐ml syringe, debubbled, stored on ice, and analyzed with a GEM 3000 analyzer (Instrumentation Laboratories) to measure P_a_O_2_, P_a_CO_2_, pH_a_, hematocrit, hemoglobin, and O_2_ saturation. The alveolar–arterial PO_2_ difference (A‐aDO_2_) was derived from respiratory data and the corresponding average of the four arterial blood gas samples. All measurements were repeated during eSVI where the mixed expired gas samples and associated arterial blood gases were collected in duplicate at the end of the last two 100% O_2_ breathing periods (providing a quadruplicate sample set), and in duplicate at the end of a 20‐min recovery air breathing period. $\dot{V}$O_2_ and respiratory exchange ratio were not calculated at the end of the eSVI because O_2_ consumption cannot be accurately measured during 100% O_2_ breathing.

Inert gas concentrations were measured in the mixed expired and arterial blood samples using gas chromatography (5890A; Hewlett‐Packard) (Wagner, Naumann, et al., 1974). Mixed venous concentrations of the inert gases were calculated by the Fick principle. Retention‐solubility and excretion‐solubility curves were generated for the six gases in each sample and transformed into a continuous plot of perfusion against ${\dot{V}}_{A}/\dot{Q}$ and ventilation against ${\dot{V}}_{A}/\dot{Q}$ using the 50‐compartment model (Wagner, Saltzman, et al., 1974). The residual sum of squares (RSS) for the measured 6‐gas data and the least squares best fit to the 50 compartment model was calculated for each data set (Hopkins & Wagner, 2017). The degree of mismatch was determined by calculating the second moments, about their mean, of the perfusion and ventilation distributions, on a log ${\dot{V}}_{A}/\dot{Q}$ scale excluding shunt and deadspace compartments, which are evaluated separately. The second moments are termed LogSD $\dot{Q}$ representing heterogeneity of the perfusion versus ${\dot{V}}_{A}/\dot{Q}$ distribution, and LogSD $\dot{V}$ representing heterogeneity of the ventilation versus ${\dot{V}}_{A}/\dot{Q}$ distribution.

### 2.4 Statistical analysis

Statistical analyses were performed using KaleidaGraph (v4.5, Synergy Software). ANOVA for repeated measures was used to evaluate differences between baseline, eSVI, and recovery for all indices derived from MIGET, ventilation, and blood gas data. Where overall significance occurred, post hoc testing was conducted using a two‐tailed Student's *t* test to evaluate differences between the three conditions. Significance was accepted at *p* < .05. All data are expressed as mean ± standard deviation.

## 3 RESULTS

### 3.1 Subject demographics

**FIGURE 2:**
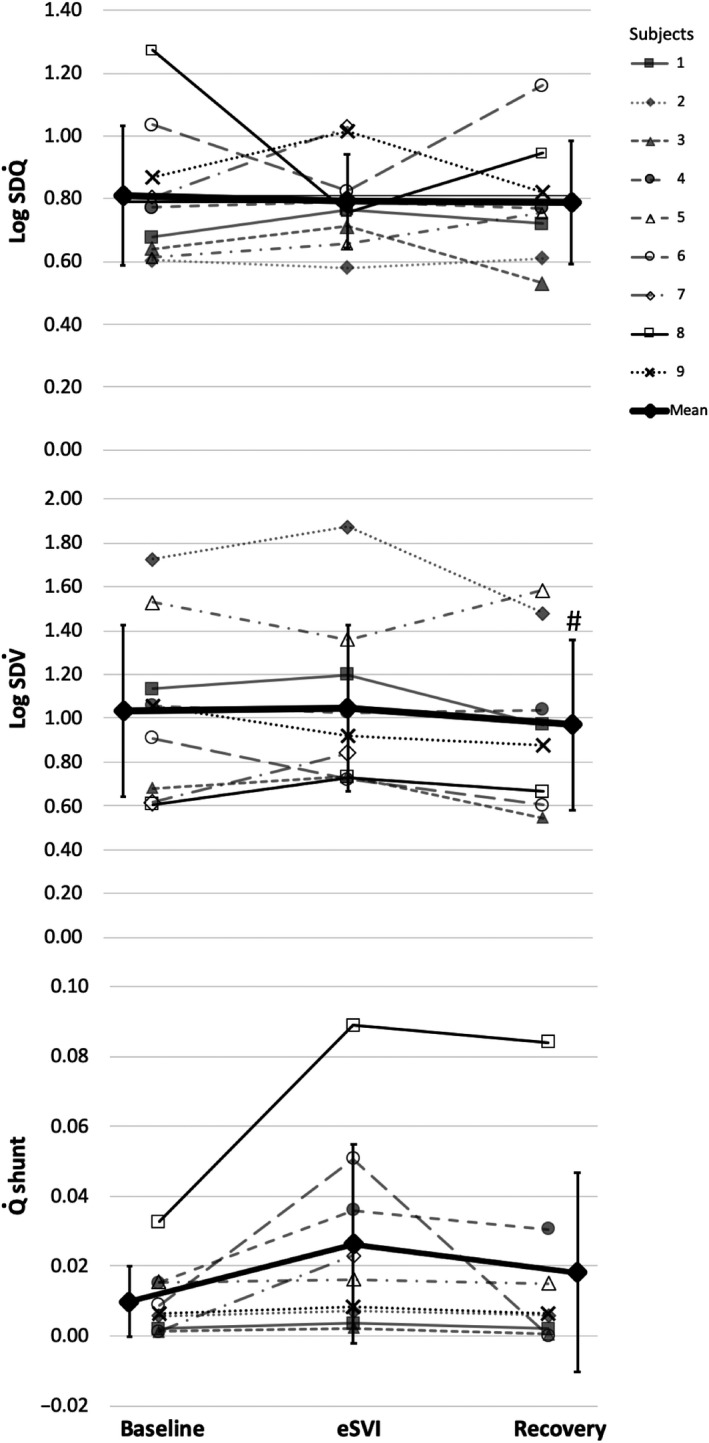
Individual LogSD $\dot{Q}$, LogSD $\dot{V}$ and shunt for the nine subjects, at baseline, eSVI, and recovery. LogSD $\dot{Q}$ = heterogeneity of the perfusion versus $V_{A}/Q$ distribution, LogSD $\dot{V}$ = heterogeneity of the ventilation versus $V_{A}/Q$ distribution; and $\dot{Q}$shunt = percent of cardiac output to shunt regions ( ${\dot{V}}_{A}/\dot{Q}$ < 0.005). ^#^denotes post hoc statistical difference between baseline and recovery at *p* < .05 (two‐tailed *t* test). Note: normal LogSD $\dot{Q}$ range of 0.3–0.6; and normal LogSD $\dot{V}$ range 0.3–0.65 (Hopkins & Wagner, 2017)

All subjects tolerated the study well. The subjects’ demographics, spirometric and blood gas data are listed in Table 1. The extent of pulmonary disease in this population ranged from very mild to severe based on spirometry with FEV_1_ percent predicted ranging from 99% to 22%. Three of the subjects (Subjects 6, 7, and 8) with self‐reported smoking histories of 37, 30, and 10 pack years, had relatively normal spirometry, despite their clinical diagnosis of COPD. These three subjects had A‐aDO_2_ differences of 35, 29, and 42 mmHg, respectively, and LogSD $\dot{Q}$ values above the normal LogSD $\dot{Q}$ range of 0.3–0.6 (Figure 2). These gas exchange values are similar to that of GOLD stage 1 COPD patients (Rodríguez‐Roisin et al., 2009).

**TABLE 1:** Baseline individual subject's demographics, spirometric data, and blood gas data

Subject	Sex	Age years	Height cm	BMI kg/m^2^	FVC % pred.	FEV_1_% pred.	FEV_1_/FVC % pred.	$\dot{V}$_E_ l /min BTPS	$\dot{V}$ O_2_l/min BTPS	P_a_O_2_ mmHg		P_a_CO_2_ mmHg	AaDO_2_ mmHg
01	M	64	178	31.8	42	22	53	9.9	311	73	44		29
02	M	57	187	20.6	50	73	69	9.2	235	77	42		24
03	M	67	178	32.8	59	52	88	7.1	254	75	45		23
04	M	70	172	28.7	74	61	82	8.1	268	74	34		38
05	M	55	158	18.0	91	75	83	7.0	165	81	39		26
06	F	49	178	33.5	99	101	100	9.7	280	73	39		35
07	M	53	159	34.8	73	81	108	10.0	292	77	42		29
08	F	66	154	34.6	92	92	98	9.9	238	83	23		42
09	M	69	178	24.3	78	60	77	6.7	221	64	34		42
Mean ± *SD*		61 ± 8	171 ± 12	28.8 ± 6.3	73 ± 20	69 ± 23	84 ± 17	8.5 ± 1.4	244 ± 40	76 ± 6	37 ± 7		32± 8

Note

*n* = 9; (mean ± *SD*).

Abbreviations: A‐aDO_2actual_, alveolar‐arterial difference for oxygen; $\dot{V}$_E_, minute ventilation; P_A_O_2_, alveolar oxygen partial pressure; FEV_1_% pred._,_ forced expiratory volume in 1 s percent predicted; FEV_1_/FVC % pred., forced expiratory volume in 1 s to forced vital capacity ratio percent predicted; FVC % pred., forced vital capacity percent predicted; P_a_CO_2_, arterial carbon dioxide partial pressure; P_a_O_2_, arterial oxygen partial pressure.

### 3.2 Metabolic, arterial blood gas data, and A‐aDO_2_ (Tables 1 and 2)

*Cardiac output,* $\dot{V}$_E_ and $\dot{V}$CO_2_ all showed no significant differences across baseline, eSVI, and recovery (*p* = .69, *p* = .22, and *p* = .61, respectively) confirming steady‐state conditions. $\dot{V}$O_2_ cannot be assessed during hyperoxic breathing and is not reported, but $\dot{V}$O_2_ was not different between baseline and recovery (*p* = .28). At baseline all nine subjects had an abnormally low P_a_O_2_ (mean P_a_O_2_ = 75.5 ± 5.7 mmHg). P_a_O_2_ showed the expected increase during eSVI, increasing to a mean P_a_O_2_ = 438 ± 40.5 mmHg (*p* < .0001) compared to baseline and recovery, and P_a_O_2_ during the recovery period (83.3 ± 7.1 mmHg) was higher than baseline (75.5 ± 5.7 mmHg) (*p* = .04). Similarly, there was the expected increase in the S_a_O_2_ from baseline (93.4 ± 1.9%) to eSVI of 95.7 ± 1.8% (*p* < .0001), and the recovery S_a_O_2_ (94.0 ± 1.9%) was also greater (*p* = .04) than baseline conditions. A‐aDO_2_ cannot be calculated during O_2_ breathing because $\dot{V}$O_2_ and respiratory exchange ratio are unknown. The baseline and recovery A‐aDO_2_ were elevated in all subjects (mean 32 ± 8 mmHg and 30 ± 12 mmHg, respectively), and were not significantly different from each other (*p* = .22). P_a_CO_2_ was not different between baseline (37.3 ± 7.0 mmHg) and eSVI (36.3 ± 6.8 mmHg), but recovery P_a_CO_2_ was significantly lower than baseline (34.6 ± 6.7 mmHg, *p* = .03), suggesting increased alveolar ventilation. There were no significant changes in pH_a_, hemoglobin or hematocrit across all three conditions, *p* = .29, *p* = .40, and *p* = .28, respectively.

### 3.3 MIGET data

The residual sum of squares (Table 2) were consistent with good data quality and adequacy of fit to the model for each condition. The RSS values were not different across conditions (*p* = .33). There were no significant differences between conditions for deadspace ventilation ( ${\dot{V}}_{\text{DS}}$) (*p* = .54) or the means (i.e., first moments) of the ventilation (*p* = .38) or perfusion distributions (*p* = .90).

**TABLE 2:** Baseline, eSVI, and recovery supine physiologic data

	Baseline	eSVI	Recovery	ANOVA *p*
Hb (g/dl)	13.7 ± 2.0	13.6 ± 2.0	13.2 ± 0.3	.40
Hct (%)	43.0 ± 7.0	41.9 ± 6.6	41.1 ± 7.1	.28
$\dot{Q}$ (l/min)	5.4 ± 0.5	5.3 ± 0.6	5.3 ± 0.5	.69
HR (bts/min)	76 ± 11	71 ± 11	73 ± 11	.03
$\dot{V}$_E_ (l/min)	8.5 ± 1.4	8.8 ± 1.4	9.0 ± 1.2	.22
$\dot{V}$O_2_ (ml/min)	244 ± 40	na	224 ± 47	.28
$\dot{V}$CO_2_ (ml/min)	206 ± 42	206 ± 44	205 ± 38	.61
RER (ratio)	0.84 ± 0.06	na	0.92 ± 0.09#	.046
S_a_O_2_ (%)	93.4 ± 1.9	95.7 ± 1.8^*^	94.0 ± 1.9^#$^	<.0001
A‐aDO_2_ (mmHg)	32.4 ± 7.7	na	29.9 ± 11.8	.22
P_a_O_2 _(mmHg)	75.5 ± 5.7	438.3 ± 40.5^*^	83.3 ± 7.1^#$^	.001
P_a_CO_2_ (mmHg)	37.3 ± 7.0	36.3 ± 6.8	34.6 ± 6.7	.03
pH_a_	7.46 ± 0.05	7.47 ± 0.05	7.47 ± 0.05	.29
RSS	5.26 ± 2.99	5.61 ± 6.22	4.40 ± 4.14	.33
$\dot{V}$_DS_ (% $\dot{V}$)	0.36 ± 0.04	0.37 ± 0.07	0.37 ± 0.09	.54
$\dot{Q}$ Shunt (% $\dot{Q}$)	1.0 ± 1.0	2.6 ± 2.9	1.8 ± 2.8	.052
Mean $\dot{V}$	1.89 ± 0.54	2.03 ± 0.50	1.89 ± 0.36	.38
LogSD $\dot{V}$	1.04 ± 0.39	1.05 ± 0.38	0.97 ± 0.39^#^	.050
Mean $\dot{Q}$	0.81 ± 0.30	0.83 ± 0.20	0.86 ± 0.24	.90
LogSD $\dot{Q}$	0.81 ± 0.22	0.79 ± 0.15	0.79 ± 0.20	.54

Note

*p* values for ANOVA testing, when significant *denotes post hoc statistical difference between baseline and emulated‐SVI; and ^#^denotes post hoc statistical difference between baseline and recovery; and ^$^denotes post hoc statistical difference between emulated‐SVI and recovery. Post hoc significance was accepted at *p* < .05 (‐tailed *t*‐test). Note: normal LogSD $\dot{Q}$ range of 0.3–0.6; and normal LogSD $\dot{V}$ range 0.3–0.65 (Hopkins & Wagner, 2017). (*n* = 9 except recovery *n* = 8, mean ± *SD*).

Abbreviations: $\dot{Q}$ shunt, percent of cardiac output to shunt regions ( ${\dot{V}}_{A}/\dot{Q}$ < 0.005); $\dot{Q}$, cardiac output; $\dot{V}$CO_2_, carbon dioxide production; $\dot{V}$_E_, minute ventilation; $\dot{V}$O_2_, oxygen consumption; A‐aDO_2_, alveolar‐arterial difference for oxygen; Hb, hemoglobin; Hct, hematocrit; HR, heart rate; LogSD $\dot{Q}$, heterogeneity of the perfusion versus ${\dot{V}}_{A}/\dot{Q}$ distribution; LogSD $\dot{V}$, heterogeneity of the ventilation versus ${\dot{V}}_{A}/\dot{Q}$ distribution; mean $\dot{Q}$, mean of the perfusion distribution; mean $\dot{V}$, mean of the ventilation distribution; na, values could not be calculated during hyperoxia; P_a_CO_2_, arterial carbon dioxide partial pressure; P_a_O_2_, arterial oxygen partial pressure; pH_a_, arterial pH; RER, respiratory exchange ratio; RSS, residual sum of squares; S_a_O_2_, arterial oxygen saturation; $\dot{V}$_DS_, percent of ventilation to deadspace ( ${\dot{V}}_{A}/\dot{Q}$ > 100).

Baseline $\dot{Q}$shunt (Table 2 and Figure 2) showed an small increase (1.0 ± 1.0% of cardiac output) compared to negligible values seen in normal subjects (Wagner, Laravuso, et al., 1974), and showed a borderline change across conditions (*p* = .052) increasing during eSVI to 2.6 ± 2.9%.

Baseline LogSD $\dot{Q}$ and LogSD $\dot{V}$ measures of heterogeneity were increased above normal values consistent with the diagnosis of COPD in these subjects (Rodríguez‐Roisin et al., 2009; Wagner, Dantzker, Dueck, Clausen, & West, 1977). Figure 2 shows baseline, eSVI and recovery LogSD $\dot{Q}$ and LogSD $\dot{V}$ and shunt values for all nine subjects. Mean LogSD $\dot{Q}$ was not significantly different across the three conditions (0.81 ± 0.22 baseline, 0.79 ± 0.15 eSVI, and 0.79 ± 0.20 recovery, *p* = .54). Mean LogSD $\dot{V}$ of 1.05 ± 0.38 during eSVI was not significantly different from baseline (1.04 ± 0.39, *p* = .84), but LogSD $\dot{V}$ in recovery showed a small but statistically significant reduction to 0.97 ± 0.39 (*p* = .04) compared to baseline, but it was not significantly different than that during eSVI (*p* = .17).

## 4 DISCUSSION

The principal findings of this study are that in subjects with varying degrees of underlying ${\dot{V}}_{A}/\dot{Q}$ mismatch and a clinical diagnosis of COPD, there was no significant change in the MIGET indices that measure global ventilation and perfusion heterogeneity, LogSD $\dot{V}$, and LogSD $\dot{Q}$ compared to baseline air breathing; the LogSD $\dot{V}$, was slightly reduced after 20 min of air breathing. There was also no significant change in deadspace, although there was a borderline increase in shunt during the eSVI protocol. These findings suggest that changes in ${\dot{V}}_{A}/\dot{Q}$ mismatch during hyperoxic exposure are small and that SVI is an appropriate method to use in proton MR measures of regional ${\dot{V}}_{A}/\dot{Q}$ ratio. However, because SVI may potentially cause small changes in pulmonary blood flow and/or shunt, we suggest the perfusion imaging always be performed before SVI so to minimize any protocol impact on ${\dot{V}}_{A}/\dot{Q}$ mapping.

### 4.1 Subject population and spirometry

In this study, baseline LogSD $\dot{Q}$ and the LogSD $\dot{V}$ measures of heterogeneity were markedly increased above normal values consistent with the goal of selecting subjects with underlying ${\dot{V}}_{A}/\dot{Q}$ mismatch and with their diagnosis of COPD (Rodríguez‐Roisin et al., 2009; Wagner et al., 1977). All subjects demonstrated hypoxemia during baseline air breathing, even subjects with very mild air flow obstruction (Table 1). The subjects with normal spirometry had significant elevation in A‐aDO_2_ and ${\dot{V}}_{A}/\dot{Q}$ mismatch (Table 1 and Figure 2), which were similar to those subjects with more severe disease. This is consistent with data from Rodríguez‐Roisin et al. (2009) who showed that the extent of ${\dot{V}}_{A}/\dot{Q}$ inequality was poorly correlated with disease severity based on spirometry.

### 4.2 Potential effects of O_2_ on the distribution of ventilation, perfusion, and ${\dot{V}}_{A}/\dot{Q}$ mismatch

The distribution of ventilation is affected by the structural heterogeneity of the bronchial and vasculature trees and the feedback systems designed to regulate the local ${\dot{V}}_{A}/\dot{Q}$ matching to maximize overall gas exchange. The respiratory gases have some regulatory control of bronchomotor tone and the degree of effect is based on the size of the affected region and local environment of the region, but the effects are small and varied (Swenson, Domino, & Hlastala, 1998). Alveolar hypoxia has a mild bronchoconstrictive effect as evidenced by increases in airway resistance (Saunders et al., 1977; Sterling, 1968), and this effect may be modulated by local CO_2_ concentrations; in general, hypocapnia enhances bronchoconstriction (Elshout, van Herwaarden, & Folgering, 1991; Ingram, 1975; Twort, Neild, & Cameron, 1985) while hypercapnia inhibits the increase in collateral airways resistance (Traystman et al., 1976). Hyperoxia exposure in healthy subjects has been shown to have little bronchomotor effect when looking at changes in maximal expiratory flow rates, airway conductances and resistances (Astin & Penman, 1967; Butler, Caro, Alcara, & Dubois, 1960; Libby et al., 1981). Consistent with this, in healthy subjects, we previously showed that neither hypoxic (F_I_O_2_ = 0.125) nor a hyperoxic (F_I_O_2_ = 0.9) (Hopkins et al., 2017) exposure altered ventilation heterogeneity as measured by the multiple breath washout.

All our subjects were hypoxemic during baseline conditions which was relieved during eSVI. In hypoxemic COPD patients, bronchoconstriction can occur (Astin & Penman, 1967; Libby et al., 1981) and is reversed by breathing 30% O_2_; but it is not known if the bronchoconstriction is the direct effect of low arterial O_2_ tensions on the bronchial smooth muscle or the reduced lower airway and alveolar O_2_ tensions. In the presence of hypoxemia‐induced bronchoconstriction, hyperoxic exposure could cause bronchodilation causing an increase in regions of high ${\dot{V}}_{A}/\dot{Q}$ ratio or deadspace ( ${\dot{V}}_{A}/\dot{Q}$ >100), but this was not seen in the present study. In some COPD patients, hyperoxia has been shown to induce hypercapnia (Abdo & Heunks, 2012; Aubier et al., 1980; Robinson et al., 2000; Sassoon et al., 1987), which is thought to be primarily caused by an increase in ${\dot{V}}_{A}/\dot{Q}$ mismatch and deadspace. This may be related to the release of hypoxic pulmonary vasoconstriction, secondary bronchodilatory effects, and a decrease in collateral resistance due to the relaxation of local parenchymal tissue associated with elevated P_a_CO_2_ due to concurrent hypoventilation. The absence of changes in ${\dot{V}}_{A}/\dot{Q}$ matching, deadspace, ventilation, or P_a_CO_2_ during hyperoxia suggests that this is not a major factor in the present study.

One possibility for the borderline increase in shunt is absorption atelectasis due to alveolar de‐nitrogenation (Dantzker, Wagner, & West, 1975). However, it is thought that parenchymal interdependence forces of attached neighboring alveoli and collateral ventilation may counteract the loss of volume and preventing alveolar collapse (Briscoe, Cree, Filler, Houssay, & Cournand, 1960; Dantzker et al., 1975). In COPD, it has been shown that the degree of collateral ventilation increases with disease severity (Hogg, Macklem, & Thurlbeck, 1969) and this increase in collateral flow potentially counteracts the development of absorption atelectasis and increase shunt (Barberà et al., 1996; Santos et al., 2000). In some COPD subjects, hyperoxia worsens ${\dot{V}}_{A}/\dot{Q}$ matching resulting in an increase in the number of low ${\dot{V}}_{A}/\dot{Q}$ units but with little or no increase in shunt (Barberà et al., 1996; Rodriguez‐Roisin, 2014; Santos et al., 2000). This is the case for the majority of subjects in the present study, and the reversibility argues against atelectasis as a mechanism. However, the response of subject #8 suggests the possibility of absorption atelectasis in this individual because the shunt did not resolve during recovery, although the changes were small.

Hyperoxia may release hypoxic pulmonary vasoconstriction resulting in a deterioration of ${\dot{V}}_{A}/\dot{Q}$ matching by increasing pulmonary blood flow to poorly ventilated or shunt regions. The time course of hypoxic pulmonary vasoconstriction during severe regional hypoxia has been shown to have different profiles based on the type of preparations or protocols, species studied, and global versus regional exposure (Sylvester, Shimoda, Aaronson, & Ward, 2012; Talbot, Balanos, Dorrington, & Robbins, 2005). In general, the hypoxic pulmonary vasoconstrictor response to global hypoxia has an initial rapid onset profile (within seconds) that reaches a maximum by 30 min, followed by a secondary gradual increase over the next 2 hr. The relaxation of the hypoxia induced vasoconstriction is less clear. In humans, 25 min of isocapnic hypoxia exposure causes a rapid rise followed by a plateau in pulmonary vascular resistance, which subsequently after 5 min of normoxia returns to baseline (Talbot et al., 2005). However, with a longer isocapnic hypoxia exposure (105 min), pulmonary vascular resistance rapidly reached a plateau then slowly continued to rise, but took longer than 10 min to return to baseline. This suggests that the relaxation response with the removal of a hypoxic stimulus may follow a different time profile than the activation process. The lack of marked changes in the present study may simply be because the hyperoxic exposure was too short (10 min in total) and intermittent.

There is evidence that structural changes, and thickening of the intimal layer of the pulmonary muscular arteries may affect the ability of the pulmonary vasculature to respond to changes in O_2_ and thus hypoxic pulmonary vasoconstriction (Barberà et al., 1994). Work in healthy subjects and in stable mild to severe COPD subjects which used MIGET before and after breathing 100% O_2_ for >30 min, (Barbera et al., 1990; Barberà et al., 1994; Wagner et al., 1977; Wagner, Laravuso, et al., 1974) showed no change in either in LogSD $\dot{Q}$, and LogSD $\dot{V}$ and no change in deadspace in healthy and some COPD subjects. However, other COPD subjects showed an increase in LogSD $\dot{Q}$, and increased perfusion to regions of low ${\dot{V}}_{A}/\dot{Q}$. This variability between COPD subjects was attributed to the degree of vascular remodeling associated with disease progression (Barberà et al., 1994). Consistent with this, two of our subjects with normal spirometry #6 and #8, (Figure 2), who had the largest increase in shunt with hyperoxia, had a conversion of existing low ${\dot{V}}_{A}/\dot{Q}$ into shunt during eSVI, which was reversed in recovery. The subjects with more severe airway obstruction (subjects 1–5, 9) showed no change in low ${\dot{V}}_{A}/\dot{Q}$ units and no increase in shunt with hyperoxia which agrees with other studies with subjects with advanced airway obstruction (Barberà et al., 1996; Santos et al., 2000). Thus, in this study, the cyclic two‐minute intermittent exposures to hyperoxia during SVI are short enough that it is unlikely that complete release of hypoxic pulmonary vasoconstriction occurs during the hyperoxia exposure. This is even less likely in COPD patients if they have lower vascular responsiveness to changes in O_2_ concentrations. Consequently, this is likely why, on average, we observed minimal changes in the LogSD $\dot{V}$ and LogSD $\dot{Q}$.

### 4.3 Post eSVI recovery and ventilation and perfusion heterogeneity

At the end of the recovery period, there was a small but significant reduction in LogSD $\dot{V}$ compared to baseline; while LogSD $\dot{Q}$ remained unchanged. The respiratory exchange ratio was significantly increased from 0.84 ± 0.06 during baseline to 0.92 ± 0.09 during recovery (*p* = .046), associated with a small decrease in PaCO_2_. This small increase in ventilation, possibly due to subject discomfort over the study course (~2 hr), may be partly responsible for the increase in P_A_O_2_ and P_a_O_2_ in recovery. The resulting ${\dot{V}}_{A}/\dot{Q}$ distribution during recovery showed a minor shift toward higher ${\dot{V}}_{A}/\dot{Q}$ units compared to baseline. Therefore, the slight improvement in gas exchange after recovery was probably due to a combination of factors, including increased ventilation, small reduction in ventilation–perfusion mismatch and return of hypoxic pulmonary vasoconstriction, none of which were statically significant on their own.

### 4.4 Limitations

MIGET is a method that measures pulmonary gas exchange by recovering the global ${\dot{V}}_{A}/\dot{Q}$ distribution from excretion and retention patterns of six infused inert gases, thus providing an overall index of ${\dot{V}}_{A}/\dot{Q}$ heterogeneity. Because of the global nature of the MIGET results, we cannot identify if there are any regional perfusion or ventilation heterogeneity changes occurring during hyperoxia that were counterbalanced by opposing changes in other regions. We studied a small number of subjects; thus, the generalizability of the findings is limited by how well the population studied is representative population as a whole. When statistical significance is lacking, the study statistical power must be considered, since a small number of subjects may impair the ability to detect meaningful differences. This study used a repeated measures design, thus each subject is their own control, and MIGET indices are highly reliable (test retest correlation ~.9 (Wagner, Hedenstierna, Bylin, & Lagerstrand, 1987)). For the observed differences in LogSD $\dot{V}$ and LogSD $\dot{Q}$ between baseline and eSVI to be statistically significant (*p* < .05, two‐tailed) in a larger sample, post hoc power calculations reveal that at least 2,338 and 228 subjects, respectively, would be required. Thus, any difference between conditions is biologically very small.

As a quality control step, MIGET retention data were matched by a fitting process, the sum of squares of the six gas residuals, and this RSS for all three conditions was calculated. The cumulative chi‐square distribution (six degrees of freedom) predicts a mean RSS of 5.3 (50th percentile) and 10.6 (90th percentile) to be indicative of an adequate data fit for the 50 compartment model (Hopkins & Wagner, 2017). Baseline, eSVI and recovery RSS were 5.3 ± 3.0, 5.6 ± 6.2, and 4.4 ± 4.1, respectively, thus demonstrating adequate data quality and supports existence of steady‐state conditions. The mean RSS for all three conditions were not statistically different from each other, suggesting that alternating F_I_O_2_ did not disturb gas exchange for MIGET gases to any identifiable degree.

## 5 CONCLUSIONS

We did not find evidence that alternating inspired O_2_ concentration between 21% and 100% for five cycles for 20 min affects ${\dot{V}}_{A}/\dot{Q}$ heterogeneity in subjects with lung disease in physiologically significant ways. Results from this study suggest that intermittent hyperoxia exposure that occurs during SVI which is used to calculate of regional ${\dot{V}}_{A}/\dot{Q}$ with proton MRI has a minimal effect on ${\dot{V}}_{A}/\dot{Q}$ heterogeneity as measured by MIGET. Accordingly, we suggest that the assumption of insignificant effects on ${\dot{V}}_{A}/\dot{Q}$ relationships due to SVI protocol is reasonable, supporting its use as part of a methodology to evaluate ${\dot{V}}_{A}/\dot{Q}$ inequality noninvasively. Nevertheless, given the findings, collection of perfusion data, which does not require altering F_I_O_2_, is recommended before performing SVI.

## CONFLICT OF INTEREST

The authors listed certify that they have no affiliations with or involvement in any organization or entity with any financial or nonfinancial interest in the subject matter or materials discussed in this manuscript.

## AUTHOR CONTRIBUTIONS

ARE, AKP, VT, GKP, PDW, and SRH contributed to study design and conception. ARE, AKP, VT, BP, JEO, HW, GKP, PDW, and SRH carried out data acquisition. All authors performed analysis and interpretation. ARE and SRH drafted the manuscript; all authors revised the manuscript. All authors provided final approval of the manuscript. ARE had full access to the data in the study and takes responsibility for the integrity of the data and the accuracy of the data analysis.
